# Identification of a PANoptosis-related gene signature reveals therapeutic potential of SFRP2 in pulmonary arterial hypertension

**DOI:** 10.3389/fcvm.2025.1521087

**Published:** 2025-04-29

**Authors:** Li Li, Mukamengjiang Juaiti

**Affiliations:** Department of Cardiovascular Medicine, The Fourth Hospital of Changsha (Changsha Hospital of Integrated Traditional Chinese and Western Medicine), Changsha, China

**Keywords:** PANoptosis, pulmonary arterial hypertension, bioinformatics, SFRP2, immune cell infiltration

## Abstract

**Background:**

Pulmonary arterial hypertension (PAH) is a serious condition marked by elevated pulmonary artery pressure, often progressing to right heart failure and high mortality. PANoptosis, an inflammatory form of programmed cell death, remains understudied in the context of PAH. This study aims to identify and validate PANoptosis-related signature genes in PAH using bioinformatics analysis alongside *in vivo* and *in vitro* experiments, seeking to uncover its potential role in disease progression.

**Methods:**

PAH-related datasets and PANoptosis-associated genes were sourced from the Gene Expression Omnibus (GEO) database and prior studies. Feature genes were identified through weighted gene co-expression network analysis (WGCNA), least absolute shrinkage and selection operator (LASSO), and random forest (RF) algorithms, with validation performed on external datasets. The immune landscape in PAH was characterized using the CIBERSORT algorithm, providing insights into immune cell composition and its role in disease progression. Gene expression was further validated using a rat PAH model and pulmonary artery fibroblasts (PAAFs), while hub gene functions were investigated at the cellular level through Western blot, CCK-8, and flow cytometry assays.

**Results:**

Through integrated transcriptomic analysis, SFRP2 was identified as a feature gene related to PAH and PANoptosis. Experimental validation was conducted in MCT-induced rat PAH models and TGF-β1-induced PAAFs, confirming SFRP2's role in regulating fibroblast proliferation and anti-apoptotic processes. The diagnostic model derived from dataset analysis exhibited high accuracy in diagnosing PAH, while immune cell infiltration analysis highlighted immune dysregulation associated with the condition.

**Conclusion:**

SFRP2 was identified as a potential biomarker for PAH, impacting cell proliferation and resistance to apoptosis, thus providing new insights for PAH prevention and treatment.

## 1 Introduction

Pulmonary arterial hypertension (PAH) is a condition characterized by elevated pulmonary artery pressure, often leading to right heart failure and other severe clinical consequences. It affects approximately 25 out of every 1 million people, with about 5 new cases emerging annually in the same population (1). Various factors, such as genetic predisposition, environmental influences, and underlying diseases, contribute to the development of PAH (2). Early symptoms, including shortness of breath, fatigue, chest pain, and palpitations, are often mild, making timely diagnosis challenging and complicating clinical decision-making (3). As a result, identifying hub genes associated with PAH could play a pivotal role in its early detection and improved management, potentially altering the course of this debilitating disease.

Cell death is integral to maintaining tissue stability, particularly during recovery from tissue injury or infection, as it removes inflammatory and stromal cells essential for tissue repair (4). This clearance process is fundamental for restoring cellular equilibrium and sustaining the structure and function of organs (5). PANoptosis, an inflammatory programmed cell death that combines pyroptosis, apoptosis, and necroptosis, plays a significant role in this homeostasis (6). Studies indicate that PANoptosis is intricately linked to various lung conditions, including acute lung injury (ALI), acute respiratory distress syndrome (ARDS), and chronic obstructive pulmonary disease (COPD) (7). These conditions frequently involve mechanisms of inflammation and tissue remodeling, which are also pertinent to PAH progression. For instance, in lung fibrosis—closely associated with PAH—an inflammatory response can prompt epithelial-mesenchymal transition (EMT) and impact the pyroptotic activity of epithelial cells (8). Furthermore, biomarkers associated with PANoptosis, such as TNFR1, NLRP3, and key proteins involved in pyroptosis, apoptosis, and necroptosis pathways, have been identified as influential factors in the inflammatory responses that drive vascular remodeling in PAH (9). During chronic inflammation or bacterial infection, pathways involving Toll-like receptors (TLRs) and other death receptors modify the activation of key proteins, including caspase-8 and RIPK1, which are critical in the inflammatory landscape of PAH (10). Research has shown that downregulating PANoptosis-related genes can alleviate inflammatory responses, suggesting a promising therapeutic avenue for PAH (9). In PAH, inflammatory macrophages release molecular signals through mechanisms such as pyroptosis, amplifying inflammation and contributing to vascular remodeling and elevated arterial pressure (11). By targeting the PANoptosome complex and modulating specific pathways of PANoptosis, there is potential to develop therapies that mitigate inflammation, slow disease progression, and alleviate vascular damage characteristic of PAH.

Bioinformatics and machine learning have become effective tools for exploring potential mechanisms and biomarkers (12). In this study, we merged two GEO datasets and integrated differentially expressed genes (DEGs) with PANoptosis-related genes to identify PANoptosis-related DEGs (PR-DEGs) for functional enrichment. We then analyzed gene correlations and expression levels using Weighted Gene Co-Expression Network Analysis (WGCNA), Least Absolute Shrinkage and Selection Operator (LASSO) logistic regression, and Random Forest (RF). A nomogram and ROC curve assessed diagnostic value. Additionally, immune infiltration analysis examined the relationship between characteristic genes and immune cell infiltration in PAH. Final hub genes were validated in two datasets, with protein expression levels confirmed in animal and cellular models, highlighting their roles in cell proliferation and anti-apoptosis. This study ultimately offers new therapeutic targets for PAH.

## 2 Materials

### 2.1 Data source

**Figure 1:**
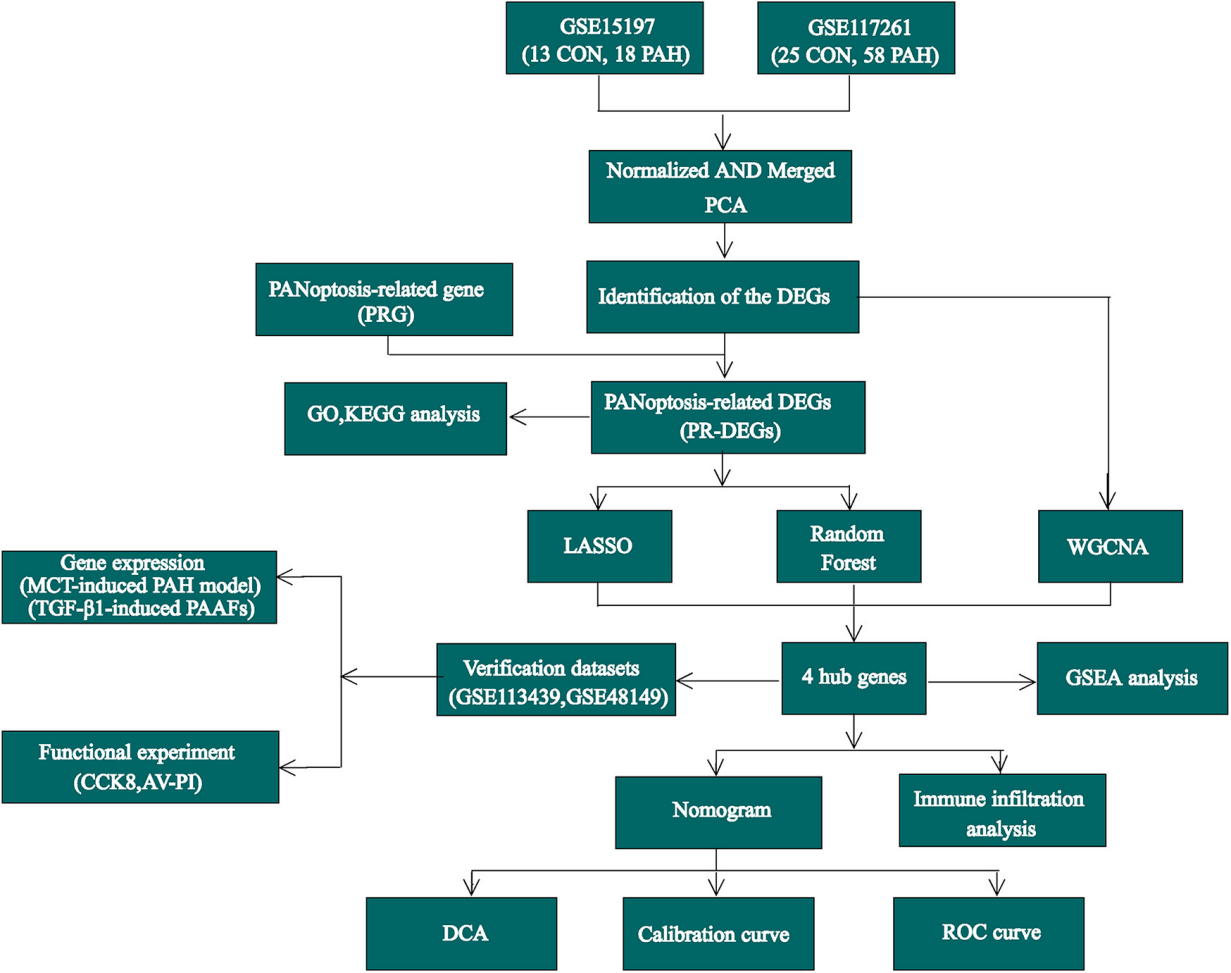
The flow chart of this study.

For this study, the gene expression datasets GSE15197 (13), GSE117261 (14), GSE113439 (15), and GSE48149 (16) were selected. All datasets were retrieved from the GEO database (https://www.ncbi.nlm.nih.gov/geo/) as standardized and quality-controlled gene expression matrices. The GSE15197 dataset includes lung tissue samples from 18 PAH patients and 13 normal controls, while GSE117261 consists of 58 PAH and 25 control lung tissue samples. The GSE113439 dataset contains lung tissue samples from 15 PAH patients and 11 control subjects, and the GSE48149 dataset was obtained from lung tissue samples, comprising 9 control subjects and 8 patients diagnosed with PAH.GSE15197 and GSE117261 were used as the training datasets, while GSE113439 and GSE48149 were selected as validation datasets. The datasets GSE113439 and GSE117261 are based on the GPL6244 platform, GSE15197 is based on the GPL6480 platform, and GSE48149 is based on the GPL16221 platform. After removing batch effects using Surrogate Variable Analysis (SVA), the GSE15197 and GSE117261 microarray datasets were integrated to form the training dataset (17). Principal component analysis (PCA) was used to visualize the differences between batches before and after SVA adjustment. Data normalization and background correction were carried out using the robust multi-array average (RMA) method. Key regulatory genes involved in apoptosis, pyroptosis, and necroptosis are classified as PANoptosis-related genes, and this gene set was collected from the previous research literature (Supplementary Table S1) (18–20). The study's analysis workflow is illustrated in Figure 1.

### 2.2 Characterization of DEGs and enrichment analysis

Raw data were processed in R software, and differential expression analysis was performed using the “limma” package (21), and DEGs between the PAH and control groups were identified based on the criteria adj.*p* < 0.05 and |log2FC| > 0.5. Visualization of DEG expression data was done using the “ggplot2” and “pHeatmap” packages, generating volcano plots and clustered Heatmaps, respectively (22). PANoptosis-related DEGs(PR-DEGs) were derived from the intersection of DEGs and PR-DEGs. Enrichment analysis of Gene Ontology (GO) terms and Kyoto Encyclopedia of Genes and Genomes (KEGG) pathways was carried out using the enrichGO and enrichKEGG functions within the “clusterProfiler” package (23). In this study, single-gene Gene Set Enrichment Analysis (GSEA) was conducted by calculating the correlation of each diagnostic gene with others, ranking all genes by correlation in descending order, and then applying GSEA through the gseGO and gseKEGG functions in the “clusterProfiler” package.

### 2.3 Weighted gene co-expression network analysis (WGCNA)

The WGCNA package in R facilitated the construction of a weighted gene co-expression network to identify modules linked to PAH. Genes with low median absolute deviation (MAD) were removed, and the pickSoftThreshold function identified an optimal soft threshold power to achieve scale-free topology (24). The resulting adjacency matrix was converted into a topological overlap matrix (TOM), followed by unsupervised clustering through hierarchical clustering to detect modules. After refinement, 12 distinct gene modules were identified, each represented by its eigengene summarizing the gene expression profile. The physiological significance of these modules was explored by correlating module eigengenes with PAH traits, and significant modules were visualized using labeled Heatmap. Modular membership (MM) and gene significance (GS) scores were then analyzed to investigate the relationships between individual genes, modules, and clinical phenotypes.

### 2.4 Machine learning

Machine learning techniques like LASSO regression and RF are frequently applied for variable selection (25, 26). LASSO, utilizing L1 regularization, serves as a linear model that supports feature selection and regression by adding an L1 penalty, which reduces certain coefficients to zero. This approach is particularly useful for handling high-dimensional data and minimizing overfitting, thus enhancing model stability. In this study, key genes were identified using a 10-fold cross-validation to determine the optimal lambda value. Random Forest, an ensemble learning method, combines predictions from multiple decision trees trained on random subsets of data to perform classification or regression tasks. This approach improves accuracy and robustness, making it well-suited for handling complex, high-dimensional, and noisy datasets. The analyses utilized the “glmnet” and “randomForest” packages in R.

### 2.5 Development of a nomogram for diagnostic evaluation

The “rms” package was utilized to build a nomogram to comprehensively assess the diagnostic value of the selected genes. A calibration curve was then plotted to evaluate the model's accuracy. Within this model, each gene was assigned a specific score, and the cumulative scores of the three identified genes were used to estimate PAH probability.

### 2.6 Assessment of diagnostic performance for signature genes and nomogram

The expression levels from the previously merged dataset were compared with those in the validation dataset. ROC curves were generated using the “pROC” package to evaluate the diagnostic performance of both the characteristic genes and the nomogram.

### 2.7 Analysis of immune cell infiltration patterns

CIBERSORT, accessible in both R and web-based formats, leverages linear support vector regression to decompose the expression matrix of diverse human immune cell subtypes. This method assesses the infiltration levels of 22 distinct immune cell types within samples based on gene expression profiles specific to each subtype. In this research, the CIBERSORT algorithm, implemented through its R script, was employed to determine the relative proportions of infiltrating immune cells across samples. The infiltration differences between the PAH group and controls were compared, with immune cell correlations visualized using the “corrplot” package. Furthermore, the associations between hub genes and immune cells were examined.

### 2.8 Single-cell analysis reveals hub gene expression in pulmonary artery cells of PAH patients

Using the publicly available GEO dataset GSE210248 (27), we conducted single-cell analysis of PAH and control groups using the Seurat package (version 4.4.0). A Seurat object was created with the following filtering criteria: min.cells = 3, min. features = 200, mitochondrial content < 20%, erythrocyte content < 0.1%, and nFeature_RNA < 3,000. Batch effects were corrected using Harmony, and the datasets were subsequently integrated. PCA and UMAP were applied for dimensionality reduction. The FeaturePlot function was used to visualize the expression of hub genes across different cell subpopulations in the pulmonary artery.

### 2.9 MCT-induced PAH rat model

The Animal Care and Use Committee of Central South University Xiangya Hospital (Changsha, China) granted approval for this study, which adhered to NIH guidelines for animal care. We employed the MCT-induced PAH model because it is one of the most classic and widely used models for studying the mechanisms of PAH and evaluating potential therapeutic agents (28, 29). Twelve male Sprague-Dawley rats (weighing 200–250 g) were kept under standard laboratory conditions and acclimated for one week before random assignment into control and MCT groups (*n* = 6 per group). The control group received an intraperitoneal injection of saline, whereas the MCT group was given monocrotaline (60 mg/kg, Sigma-Aldrich, USA) to induce PAH. The study period was 21 days, with the rats housed in a pathogen-free setting. At the end of the experiment, the rats were euthanized, and lung tissues were collected for histological examination and Western blot analysis. Right ventricular hypertrophy was evaluated by determining the ratio of right ventricular weight to the combined weight of the left ventricle and septum.

### 2.10 H&E staining

Lung tissue samples were fixed, embedded in paraffin, and sectioned into slices of 5 μm thickness. Hematoxylin and eosin (HE) staining was performed on these sections using a kit (C0105S, Beyotime, China) in accordance with the manufacturer's instructions. Following staining, the sections were mounted and examined microscopically for analysis.

### 2.11 Real-time PCR

All rats were euthanized under anesthesia, and their lung tissues were collected for total RNA extraction using the TRIzol reagent (R0016, Beyotime, China). Complementary DNA was synthesized, and quantitative real-time PCR (qRT-PCR) was performed using the SYBR Green One-Step qRT-PCR Kit (D7268S, Beyotime, China) to assess the RNA expression levels of specific target genes. The primer sequences used in this study are listed in Supplementary Table S2.

### 2.12 Western blot

Proteins were extracted using RIPA buffer (YSD0100, Yoche, China) supplemented with 1% PMSF (G2008-1ML, Servicebio, China). The protein concentration was measured with a BCA kit (YSD-500T, Yoche, China). Samples were separated on SDS-PAGE gels and transferred to PVDF membranes. After blocking, the membranes were incubated overnight at 4°C with primary antibodies: SFRP2 (1:1,000, GB11880, Servicebio), PCNA (1:5,000, GB11010, Servicebio), BCL2 (1:1,000, GB154380, Servicebio), and BAX (1:1,000, GB154122, Servicebio). Internal control was performed with α-Tubulin (1:5,000, GB15200, Servicebio). Following three 10-minute washes, secondary antibodies (1:10,000, GB23303 and GB2330, Servicebio) were applied at 37°C for 1 h. The bands were visualized using ECL solution (PYT005, Yoche, China) and analyzed with Gel-ProAnalyzer software after additional washing steps.

### 2.13 Cell culture experiment

Primary pulmonary artery adventitial fibroblasts (PAAFs) from rats were isolated and cultured following a previously established method (30). In summary, rats were euthanized under anesthesia, and lung tissue was quickly excised. The distal pulmonary artery was carefully dissected to isolate the adventitial layer, and connective tissue was removed to expose fibroblasts. The adventitial layer was then finely minced and digested in HBSS containing 1 mg/ml collagenase I (Sigma-Aldrich, USA) at 37°C for 20–30 min to release PAAFs. The cell suspension was filtered and centrifuged, and cells were cultured in DMEM/F12 supplemented with 10%–20% FBS until confluent. For experiments, 3rd to 5th-generation PAAFs at 70%–80% confluence were used. To stimulate PAAFs, cells were serum-starved in 0.5% FBS medium for 24 h to synchronize cell cycles, preparing them for TGF-β1 induction. After serum starvation, PAAFs were treated with TGF-β1 (Peprotech, USA) at 10 ng/ml for 24 h to promote fibroblast activation and differentiation. For gene knockdown, PAAFs were transfected with either control siRNA or si-SFRP2 following the manufacturer's protocol. The siRNA, synthesized by Ribobio (China), had the sequence 5′-CACTGTAAATATTTCAGATAAAC-3′ for si- SFRP2.

### 2.14 Cell counting Kit-8 (CCK-8) assay for cell proliferation

The Cell Counting Kit-8 (CYT001-500, Qichun, China) assay was employed to assess PAAFs proliferation. In brief, pretreated PAAFs were exposed to TGF-β1 for designated time periods. Subsequently, 10 μl of CCK-8 solution was added to each well, followed by a 2 h incubation at 37°C in a humidified incubator. Absorbance was then measured at 450 nm using a microplate reader to evaluate cell proliferation rates.

### 2.15 Annexin V-Pi dual staining for apoptosis detection by flow cytometry

Cells were seeded in a 6-well plate and divided into control, single-positive, and experimental groups (Control, TGF-β1 + NC, TGF-β1 + si-SFRP2. After appropriate transfection and treatment, cells were collected, digested, and washed in PBS. Experimental groups were stained with Annexin V-FITC and PI, incubated in the dark for 10 min, and analyzed within 1 h. Flow cytometry was performed at 488 nm, detecting FITC fluorescence on FL1 and PI on FL3. Data analysis was conducted using FlowJo with a cross gate to identify positive signals.

### 2.16 Statistical analysis

Data are presented as mean ± SD. Most comparisons were analyzed using one-way ANOVA with Tukey's *post hoc* test, while an unpaired Student's *t*-test was applied where appropriate. Statistical significance was set at *p* < 0.05. All analyses were conducted using R software (version 4.2.1) and GraphPad Prism software (version 8.0).

## 3 Results

### 3.1 Identification and enrichment analysis of DEGs in PAH

**Figure 2:**
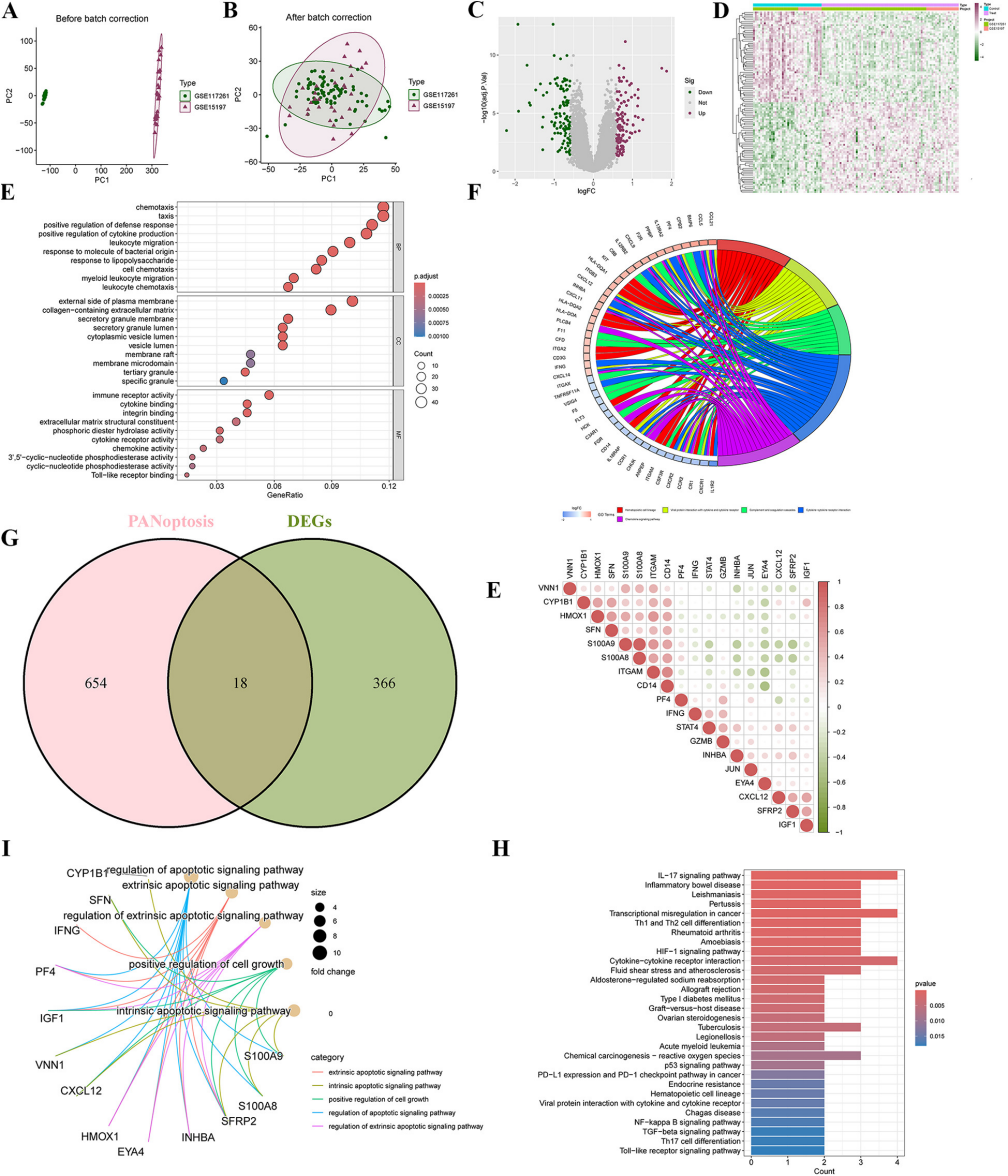
Screening differentially expressed genes (DEGs) and PANoptosis-related DEGs (PR-DEGs) in pulmonary arterial hypertension (PAH). **(A)** Principal Component Analysis (PCA) illustrates a distinct separation between the PAH and control groups in the merged datasets of GSE15197 and GSE117261. **(B)** The volcano plot highlights the upregulated (red) and downregulated (green) DEGs. **(C)** A clustering analysis with an accompanying Heatmap depicts the expression profiles of DEGs between the PAH and control groups. **(D)** Bubble plots visualize the GO enrichment analysis of DEGs, encompassing biological processes, cellular components, and molecular functions. **(E)** The circus plot presents the KEGG enrichment analysis of DEGs. **(F)** A Venn diagram identifies the overlapping DEGs shared with PANoptosis-related genes. **(G)** The Heatmap displays the correlations among PANoptosis-related DEGs. **(H,I)** GO and KEGG enrichment analyses of PR-DEGs are visualized, offering insights into their biological significance. GO, gene ontology; KEGG, Kyoto encyclopedia of genes and genomes.

To illustrate the batch differences before and after adjustment between GSE117261 and GSE5197, a two-dimensional PCA clustering plot was generated. The analysis revealed that once batch effects were resolved, the two sample groups displayed clear clustering (Figure 2A). A total of 384 differentially expressed genes (DEGs) were identified, comprising 197 upregulated and 187 downregulated genes, as illustrated in the volcano plot (Figure 2B) and Heatmap (Figure 2C). GO and KEGG enrichment analyses were performed to investigate the biological functions and signaling pathways related to these DEGs. GO analysis revealed that DEGs were significantly enriched in biological processes (BP) such as chemotaxis, taxis, positive regulation of defense response, and cytokine production; cellular components (CC) including the “external side of plasma membrane” and “collagen-containing matrix metalloproteinase”; and molecular functions (MF) like “immune receptor activity” and “cytokine binding” (Figure 2D). KEGG analysis indicated significant enrichment of DEGs in pathways such as “Hematopoietic cell lineage”, “Cytokine-cytokine receptor interaction”, and the “Chemokine signaling pathway” (Figure 2E).

### 3.2 Identification and enrichment analysis of PR-DEGs

By intersecting the 384 DEGs with 672 PANoptosis-related genes, a total of 18 PR-DEGs were identified (Figure 2F). Pearson correlation analysis indicated significant interactions among PR-DEGs (Figure 2G). GO analysis revealed significant enrichment of these PR-DEGs in pathways such as the “extrinsic apoptotic signaling pathway”, “intrinsic apoptotic signaling pathway”, “positive regulation of cell growth”, “regulation of apoptotic signaling pathway”, and “regulation of extrinsic apoptotic signaling pathway” (Figure 2H). KEGG analysis demonstrated the enrichment of PR-DEGs in pathways like the “IL-17 signaling pathway”, “HIF-1 signaling pathway”, “p53 signaling pathway”, “NF-kappa B signaling pathway”, “TGF-beta signaling pathway”, and “Toll-like receptor signaling pathway” (Figure 2I). These findings suggest that studying PANoptosis in PAH is of critical importance for understanding the pathogenesis of PAH and exploring potential therapeutic approaches.

### 3.3 Identification of PAH-associated gene modules

**Figure 3:**
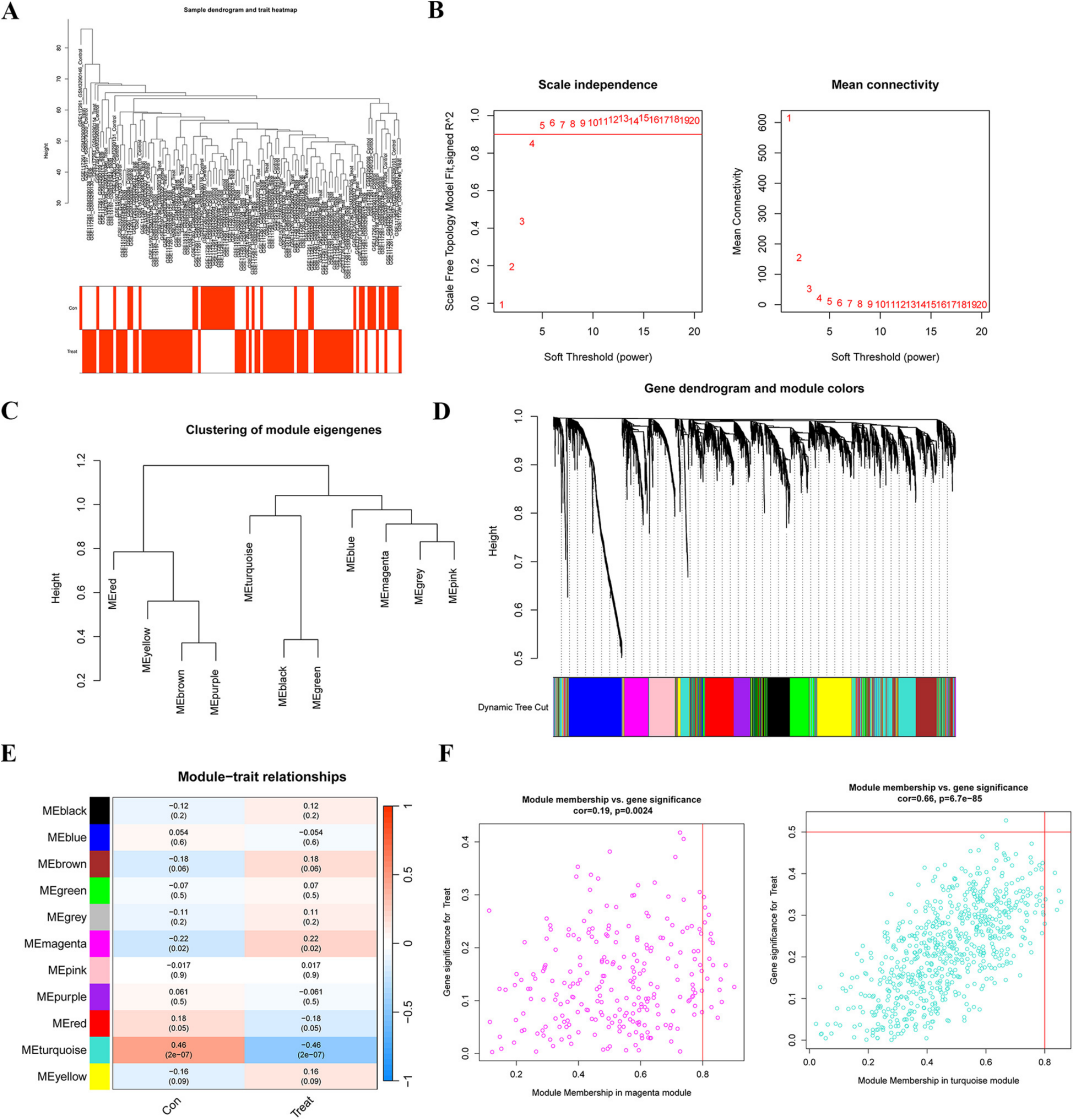
Identification of gene modules associated with PAH using WGCNA. **(A)** Sample clustering and phenotypic information for the merged dataset. **(B)** Determination of the optimal soft-thresholding power through the analysis of the scale-free fit index (left) and mean connectivity (right) across different soft-thresholding powers. **(C)** Cluster dendrogram depicting module eigengenes. **(D)** Gene dendrogram with corresponding modules, where gene modules associated with PAH are represented in distinct colors beneath the dendrogram. **(E)** Correlation Heatmap illustrating the relationships between various gene modules and PAH status. **(F)** Scatter plots displaying the correlation between module membership (MM) and gene significance (GS) in the magenta and turquoise modules. WGCNA, weighted gene co-expression network analysis.

WGCNA was used to identify gene modules significantly linked to PAH. The sample clustering dendrogram and the corresponding clinical trait Heatmap are presented in Figure 3A. A soft-threshold power of 4 was chosen as the optimal value (*R*^−2^ = 0.85) based on the scale-free topology fit index and mean connectivity, allowing for the construction of a scale-free network **(**Figure 3B). After merging modules with highly correlated eigengenes, 12 gene modules were ultimately identified **(**Figures 3C,D), and module-trait relationships were assessed and visualized (Figure 3E). The magenta module (*r* = −0.22, *p* = 0.02) and turquoise module (*r* = 0.46, *p* = 2 × 10^−07^) exhibited the highest and most significant correlations with PAH. Additionally, in the magenta module (*r* = 0.19, *p* = 0.0024) and turquoise module (*r* = 0.66, *p* = 6.7 × 10^−85^), module membership (MM) showed a significant correlation with gene significance (GS) (Figure 3F), underscoring the module's importance. Finally, we selected genes from the turquoise module, which displayed the strongest correlation and highest statistical significance, as key PAH-related genes for further analysis.

### 3.4 Screening of hub genes with diagnostic value via machine learning

**Figure 4:**
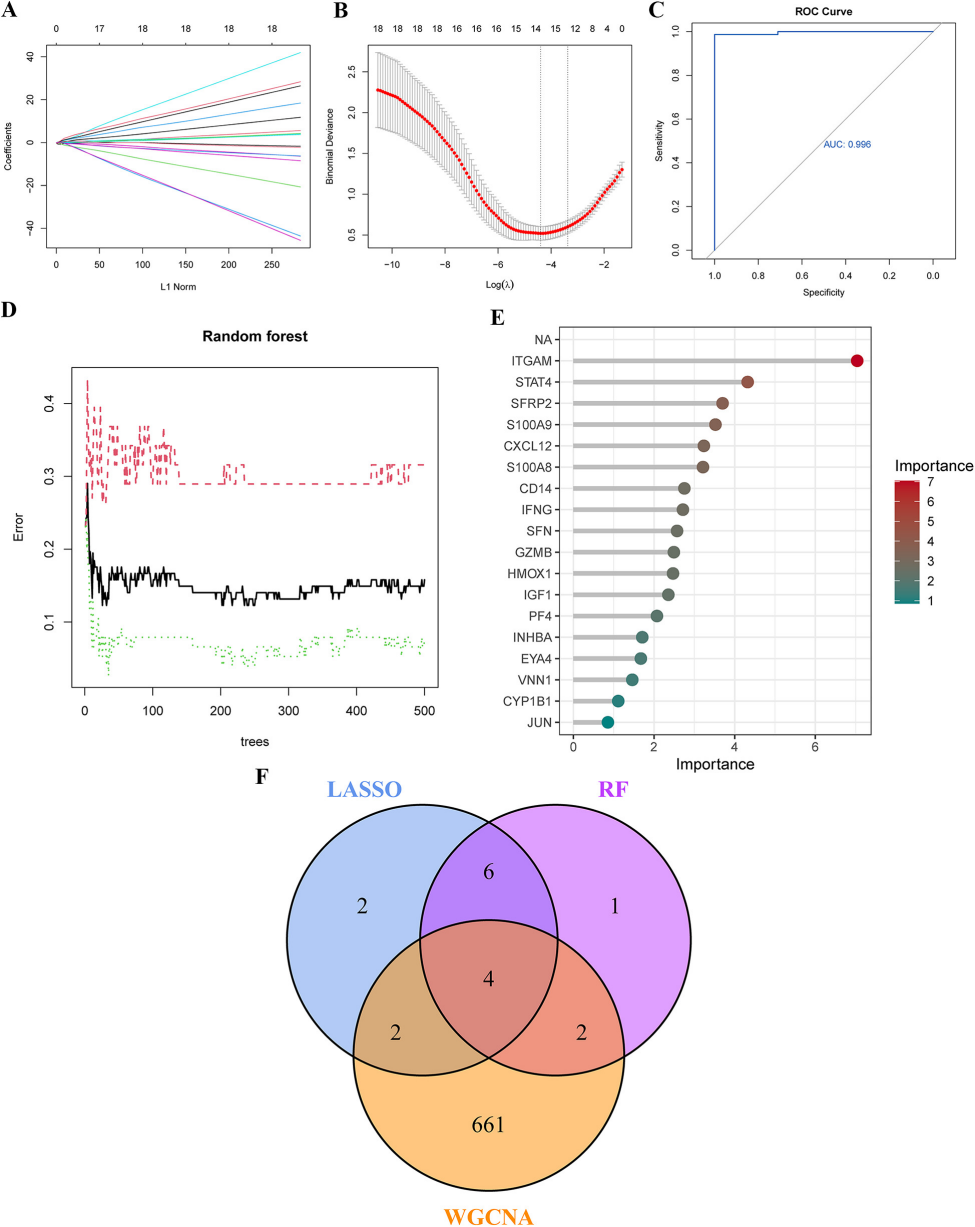
Identification of Key genes in PAH using machine learning. **(A,B)** Key genes were identified through LASSO regression analysis, with 14 genes selected as optimal candidates based on the lowest point of the binomial deviance curve. **(C)** The ROC curve derived from the LASSO model demonstrates excellent diagnostic performance. **(D)** Diagnostic errors were visualized using the random forest (RF) model. **(E)** In the RF model, genes were ranked in descending order based on their MeanDecreaseGini values, indicating their importance. **(F)** A Venn diagram shows four overlapping genes identified by the LASSO model, RF algorithm, and WGCNA. LASSO, least absolute shrinkage and selection operator; ROC curve, receiver operating characteristic curve.

To screen for hub genes with diagnostic value, we applied LASSO and RF machine learning algorithms to identify key PAH- and PANoptosis-associated genes from the 18 PR-DEGs. In the LASSO regression analysis, set to a binomial family, cross-validation generated gene coefficient and binomial deviance plots (Figures 4A,B). Hub genes were selected based on variables linked to the optimal penalty parameter with the best lambda value (lambda.min = 0.012). The ROC curve demonstrated strong diagnostic performance for this model (Figure 4C). Fourteen hub genes were ultimately identified through LASSO regression, including ITGAM, S100A8, CD14, SFRP2, INHBA, EYA4, CXCL12, GZMB, VNN1, IGF1, PF4, SFN, IFNG, and CYP1B1.In the RF algorithm, diagnostic errors were visualized, and candidate genes were ranked by variable importance (Figures 4D,E). Genes with a MeanDecreaseGini of 4 were identified as significant, comprising ITGAM, STAT4, SFRP2, S100A9, CXCL12, S100A8, CD14, IFNG, SFN, GZMB, HMOX1, IGF1, and PF4. By intersecting the top genes from LASSO, RF, and WGCNA, four characteristic genes (ITGAM, S100A8, CD14, and SFRP2) were identified (Figure 4F).

### 3.5 Single-gene GSEA of characteristic genes

**Figure 5:**
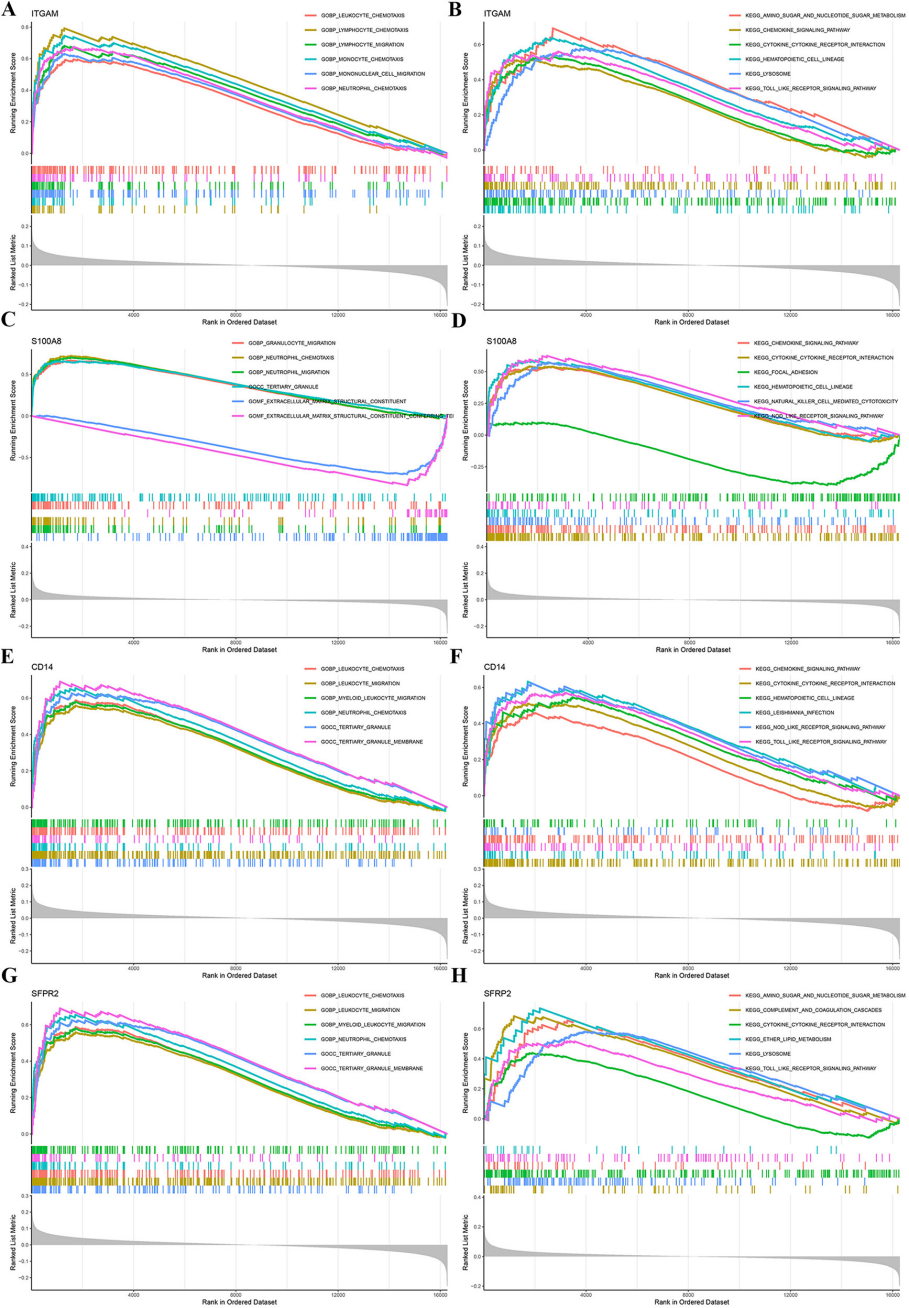
Single-gene GSEA of characteristic genes. GO and KEGG enrichment analyses were conducted using GSEA for the genes ITGAM, S100A8, CD14, and SFRP2 to explore their biological roles and associated pathways. GSEA, gene set enrichment analysis.

To further investigate the biological functions and pathways associated with the key genes (ITGAM, S100A8, CD14, and SFRP2), we performed single-gene GSEA, utilizing GO and KEGG enrichment analyses for each gene. The top six terms from GO and KEGG are displayed in Figures 5A–H. These analyses revealed that the characteristic genes are involved in biological processes such as leukocyte chemotaxis, lymphocyte chemotaxis, lymphocyte migration, monocyte chemotaxis, mononuclear cell migration, neutrophil chemotaxis, granulocyte migration, extracellular matrix(ECM) structural constituent, and tertiary granule formation. KEGG pathway analysis highlighted terms such as lysosome, toll-like receptor signaling pathway, amino sugar and nucleotide sugar metabolism, hematopoietic cell lineage, focal adhesion, and natural killer cell-mediated cytotoxicity. The single-gene GSEA results indicate that these key genes likely play a critical role in modulating immune response, facilitating cellular migration, and influencing various cell signaling pathways.

### 3.6 Construction of the diagnostic nomogram for PAH

**Figure 6:**
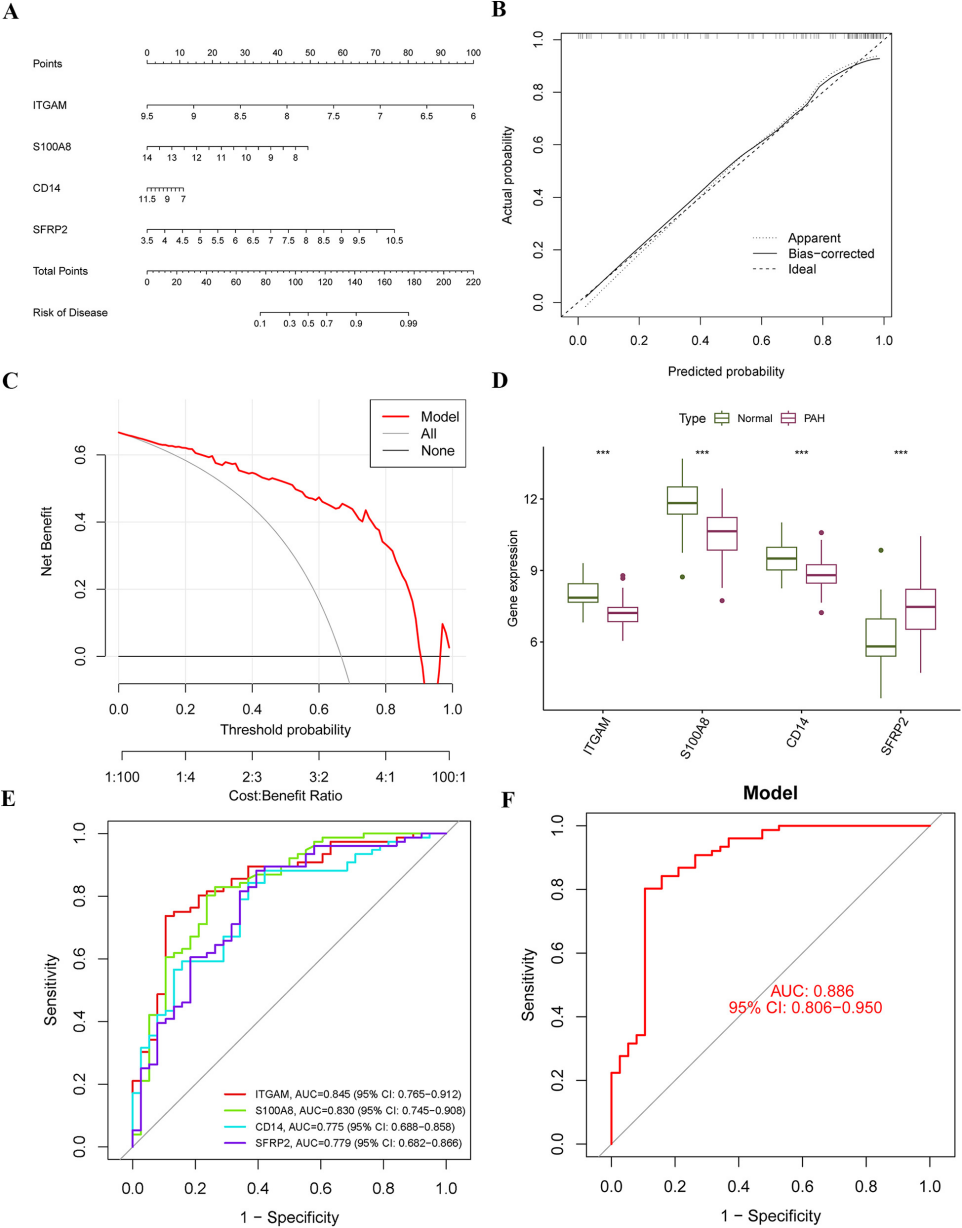
Construction of the diagnostic nomogram and performance assessment. **(A)** A diagnostic nomogram was developed based on four characteristic genes. Each gene was assigned a corresponding score, and the total score was used to predict the risk of PAH. **(B)** A calibration curve was generated to assess the accuracy and reliability of the nomogram. **(C)** Decision curve analysis (DCA) was performed to evaluate the net benefit of the nomogram in predicting PAH. **(D)** Expression levels of the four hub genes were compared within the merged dataset. **(E,F)** ROC curves were plotted to evaluate the predictive performance of the individual genes and the nomogram in diagnosing PAH.

A diagnostic nomogram for PAH, incorporating the identified characteristic genes, was developed using the “rms” package. In this nomogram, each gene is assigned a specific score, and the cumulative score of the four genes is utilized to estimate the risk of PAH (Figure 6A). The calibration curve demonstrates that the nomogram's predicted probabilities closely align with those of an ideal model (Figure 6B), and decision curve analysis indicates that decisions based on the nomogram are beneficial (Figure 6C). Expression analysis in the training cohort reveals that ITGAM, S100A8, and CD14 are significantly downregulated in PAH, while SFRP2 is significantly upregulated (Figure 6D). All four hub genes exhibit strong diagnostic performance, with area under the ROC curve (AUC) values of 0.845, 0.830, 0.775, and 0.779 for ITGAM, S100A8, CD14, and SFRP2, respectively (Figure 6E). The nomogram based on these four hub genes shows robust diagnostic efficacy, with an AUC value of 0.886 (Figure 6F).

### 3.7 Immune cell infiltration analysis

**Figure 7:**
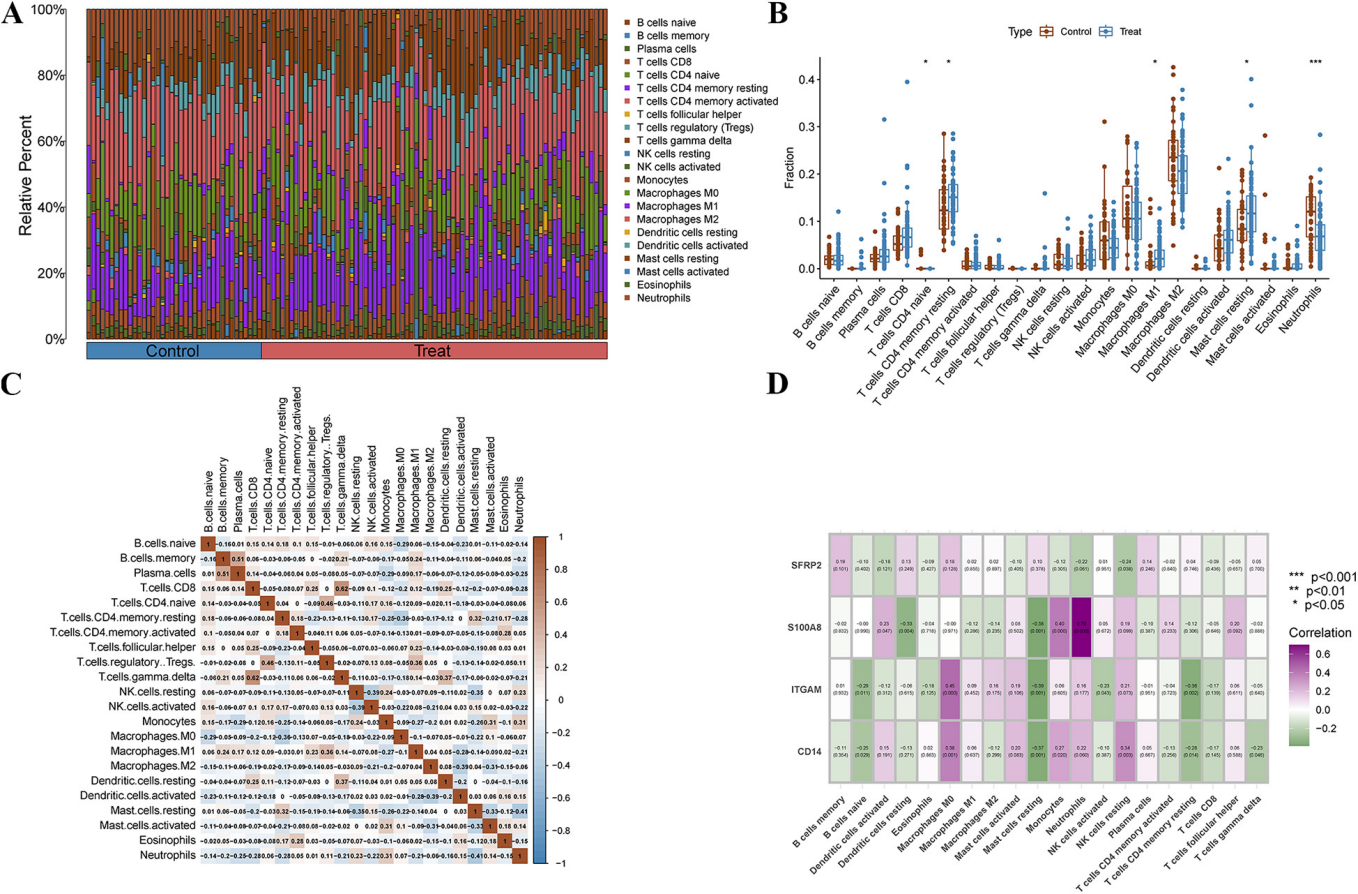
Immune cell infiltration analysis. **(A)** The stacked bar plot represents the different immune cell proportions in each sample. **(B)** The boxplot depicts the comparison of 22 types of immune cells between PAH and control groups. **(C)** The Heatmap shows the correlation between different immune cells. **(D)** The correlation Heatmap exhibits the association of the immune cells with the four characteristic genes.

We used the CIBERSORT algorithm to assess the characteristics of immune cell infiltration in PAH. Figure 7A shows the proportions of 22 immune cell types in each sample. Compared to the control group, T cell CD4 naive and neutrophils significantly decreased, while T cells CD4 memory activated, macrophages M1, and mast cells resting significantly increased (Figure 7B). Correlation analysis revealed positive correlations between B cells memory and plasma cells, T cells gamma delta and T cells CD8, and T cells regulatory (Tregs) and T cells CD4 naive. Additionally, negative correlations were observed between mast cells resting and neutrophils, mast cells activated and dendritic cells activated, and dendritic cells activated and macrophages M2 (Figure 7C). We further explored the correlation between immune cells and the four diagnostic biomarkers, as well as the association between the four hub genes and immune cells (Figure 7D). Interestingly, we found that S100A8 was positively correlated with neutrophils, SFRP2 was negatively correlated with resting NK cells, ITGAM was positively correlated with macrophages M0, and CD14 was positively correlated with macrophages M0.

We used the CIBERSORT algorithm to assess the immune cell infiltration landscape in PAH. Figure 7A shows the relative proportions of 22 immune cell types in each sample. Compared to the control group, the PAH group exhibited a significant decrease in CD4 naïve T cells and neutrophils, while CD4 memory activated T cells, M1 macrophages, and resting mast cells were significantly increased (Figure 7B), suggesting a shift from innate to adaptive immune activation, and increased pro-inflammatory responses. Correlation analysis (Figure 7C) revealed specific immune cell interaction patterns. For instance, positive correlations between memory B cells and plasma cells, or between CD8 T cells and gamma delta T cells, may reflect coordinated adaptive immune activation. Negative correlations, such as between resting mast cells and neutrophils or between activated dendritic cells and macrophages M2, suggest potential immune regulatory dynamics or cellular competition within the inflammatory microenvironment. To further explore the relationship between key diagnostic markers and immune context, we analyzed correlations between the four hub genes (SFRP2, S100A8, ITGAM, CD14) and immune cell subsets (Figure 7D). S100A8 showed strong positive correlation with neutrophils, consistent with its known role in neutrophil activation and chemotaxis. SFRP2 was negatively correlated with resting NK cells, which may imply a potential suppressive effect on innate immune surveillance. ITGAM and CD14 were both positively correlated with M0 macrophages, highlighting their potential role in modulating macrophage recruitment or differentiation in PAH. These findings suggest that the identified diagnostic markers are not only altered at the gene expression level but may also be involved in reshaping the immune microenvironment in PAH.

### 3.8 External validation of the characteristic genes and nomogram

**Figure 8:**
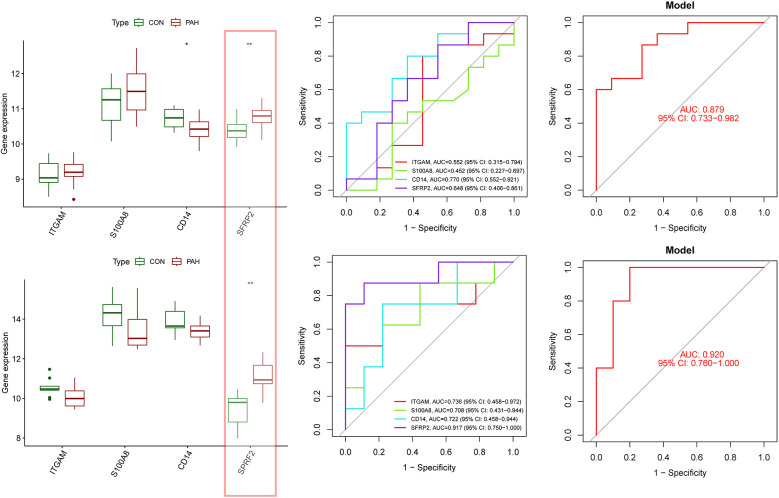
Evaluation of four characteristic genes in external datasets. **(A)** Expression levels of the four characteristic genes were compared between the PAH and control groups in the validation dataset GSE113439. **(B)** ROC curves were generated to assess the diagnostic performance of the four genes and the nomogram in GSE113439. **(C)** ROC curves demonstrated the diagnostic performance of three genes and the nomogram in GSE113439. **(D)** Expression levels of the four genes were compared in the validation dataset GSE42955. **(E)** ROC curves were used to evaluate the diagnostic performance of three genes and the nomogram in the GSE48149 dataset. **(F)** ROC curves demonstrated the diagnostic performance of three genes and the nomogram in GSE42955. Statistical significance: *p < 0.05; **p < 0.01.

The expression levels and diagnostic accuracy of the four characteristic genes were further validated using two external datasets (GSE113439 and GSE48149). In both the training and validation groups, only one hub gene, SFRP2, consistently showed significantly upregulated expression (Figures 8A,D). In the GSE113439 dataset, ROC analysis indicated that only CD14 and SFRP2 exhibited favorable diagnostic efficiency, with AUC values of 0.770 for CD14 and 0.818 for SFRP2 (Figure 8B). In the GSE48149 dataset, SFRP2 demonstrated an AUC value of 0.850 (Figure 8E). The nomogram based on the four hub genes exhibited strong diagnostic efficacy, with AUC values of 0.879 and 0.920, respectively, in the two datasets (Figures 8C,F). Therefore, we selected SFRP2 as our final hub gene.

### 3.9 Validation of SFRP2 expression and functional role in PAH fibroblast model

**Figure 9:**
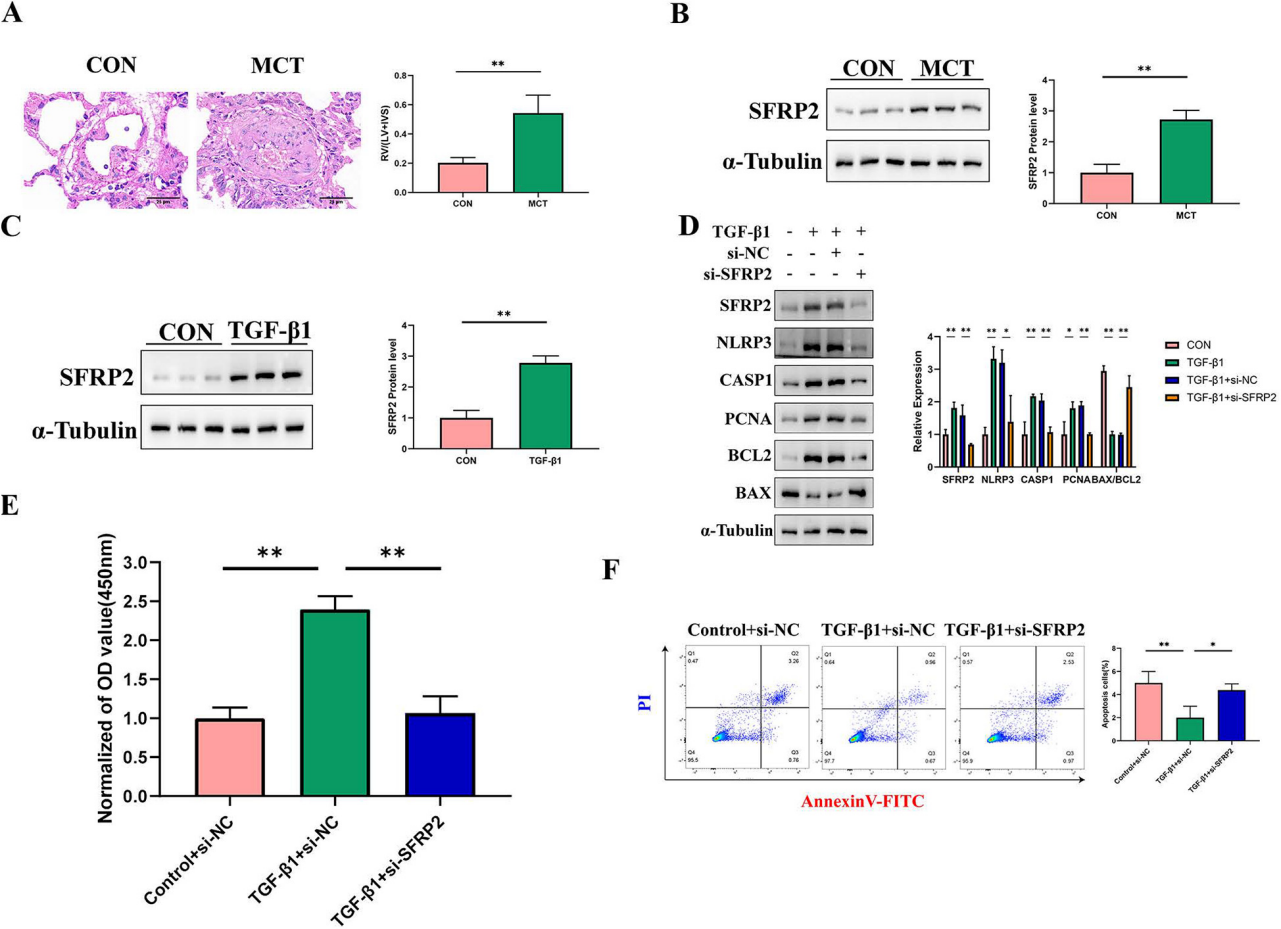
Validation of Hub genes both *in vivo* and *in vitro*. **(A)** HE staining and right ventricular hypertrophy index (RVHI) were analyzed in lung tissues from the control and MCT groups. The red color represents the control group (*n* = 6), and the green represents the MCT group (*n* = 6). RVHI was calculated as the ratio of right ventricular weight to the combined weight of the left ventricle and interventricular septum (RV/LV + S). The scale bar in the image is 25 μm. **p < 0.01. **(B)** The expression of SFRP2 in lung tissues was compared between the control and MCT groups. The left panel shows Western blot results, while the right panel displays quantitative analysis. **(C)** SFRP2 expression in PAAFs induced by TGF-β1 was examined. The left panel presents Western blot results, and the right panel provides quantitative analysis, *n* = 3 in each group. **(D)** The effect of SFRP2 knockdown on cell proliferation and apoptosis was analyzed. Compared to the control group, SFRP2 knockdown significantly enhanced the proliferation and anti-apoptotic properties of PAAFs. The left panel shows Western blot results, while the right panel displays quantitative analysis, *n* = 3 in each group. **(E)** The impact of SFRP2 knockdown on cell proliferation activity was assessed. **p < 0.01. **(F)** Annexin V-PI double staining was performed to detect apoptosis rates. Q3 represents the proportion of early apoptotic cells, and Q2 represents late apoptotic cells. The sum of Q3 and Q2 was calculated and statistically analyzed. *p < 0.05.

We first established an animal model of PAH induced by MCT. Using HE staining, we observed marked vascular thickening and remodeling in the pulmonary vessels of the MCT group. The RV/(LV + IVS) ratio in the MCT group was significantly higher than in the control group, confirming successful model establishment (Figure 9A). At the mRNA level, we used qRT-PCR to examine the expression levels of ITGAM, S100A8, CD14, and SFRP2. Our results showed significant differences in the mRNA expression of the latter three genes, while ITGAM showed no notable change (Supplementary Figure S1). At the protein level, we validated the expression of SFRP2, with Western blot results showing significantly higher SFRP2 levels in the MCT group compared to the control group (Figure 9B). Previous studies have reported SFRP2 expression in the pulmonary artery adventitia, leading us to select pulmonary artery fibroblasts as the study subject (31). Furthermore, our single-cell sequencing results revealed that SFRP2 is highly expressed in fibroblasts, further supporting its critical role in pulmonary vascular remodeling and fibrosis (Supplementary Figure S2). Results showed a marked increase in SFRP2 protein levels in fibroblasts induced by TGF-β1 (Figure 9C). Figure 9D shows that SFRP2 knockout can reverse TGF-β1-induced changes in the proliferation marker PCNA, the BAX/BCL2 ratio, as well as the expression levels of inflammasome-related proteins NLRP3 and cleaved Caspase-1. For further validation, CCK8 assay results (Figure 9E) demonstrated that SFRP2 knockout can reverse TGF-β1-induced proliferation. Additionally, flow cytometry analysis (Figure 9F) revealed that SFRP2 knockout can counteract TGF-β1-induced apoptosis resistance.

## 4 Discussion

Current research suggests that the process of cell death may serve as a critical therapeutic target in PAH (32). In PAH, when cells develop resistance to apoptosis, a dynamic molecular network is formed, allowing these cells to evade mechanisms that would typically limit their survival and disease progression (33). In this context, PANoptosis—a mechanism composed of interconnected forms of cell death—may play a unique role in the pathology of PAH. Our study establishes a robust evaluation system by leveraging bioinformatics to identify and validate key genes and molecular pathways associated with PANoptosis in PAH. Through the integration of machine learning algorithms and immune infiltration analysis, the study enhances scientific rigor and credibility, offering insights into the pathological processes and molecular mechanisms underlying PAH. This approach provides a foundation for potential targets in future clinical treatments.

In our study, we identified 384 DEGs and conducted GO and KEGG enrichment analyses, which revealed their involvement in key immune and inflammatory pathways, such as cytokine production and chemokine signaling. For instance, the chemokine signaling pathway plays a critical role in immune cell migration to specific tissue sites. In PAH, overexpression of chemokines may lead to excessive immune cell accumulation around pulmonary arteries, exacerbating inflammation, vascular smooth muscle proliferation, and remodeling (34, 35). Targeting this pathway could potentially mitigate immune cell recruitment and reduce inflammation. Similarly, the cytokine-cytokine receptor interaction pathway may sustain chronic inflammation, accelerating vascular damage and remodeling, thus worsening PAH progression (36). Strategies inhibiting specific cytokines or receptors could help alleviate these effects. The immune differential analysis highlights the involvement of multiple immune cells in the progression of PAH. A significant decrease in T cells CD4 naive, which are critical for initiating adaptive immunity, suggests compromised immune surveillance and a shift toward chronic immune activation (37). In contrast, the altered levels of T cells CD4 memory resting indicate a disturbance in immune homeostasis, potentially contributing to persistent inflammation (38). Moreover, macrophages M1, known for their pro-inflammatory phenotype, were found to be significantly elevated. These cells are recognized for their secretion of cytokines that exacerbate endothelial dysfunction, promote vascular remodeling, and amplify inflammatory responses (39). Resting mast cells also appear to play a role, as their activation releases histamine and other inflammatory mediators, which may further exacerbate vascular inflammation and fibrosis (40). Neutrophils contribute to PAH pathogenesis by releasing reactive oxygen species and neutrophil extracellular traps, mechanisms that promote endothelial damage, smooth muscle proliferation, and ECM remodeling (41).

Through screening the intersection of 384 DEGs with PANoptosis-related genes, 18 PR-DEGs were identified. GO analysis revealed significant enrichment of DEGs in apoptotic signaling pathways and cell growth regulation, while KEGG analysis highlighted pathways such as IL-17, HIF-1, p53, NF-kappa B, TGF-beta, and Toll-like receptor signaling. These results underscore the importance of PANoptosis in understanding PAH pathogenesis and identifying potential therapeutic targets. The core genes identified, including ITGAM, S100A8, CD14, and SFRP2, are closely associated with multiple inflammatory pathways and act as key genes within these pathways, suggesting that PANoptosis may be involved in the complex immune-inflammatory regulation process underlying PAH pathogenesis. Among them, ITGAM plays a vital role in immune cell adhesion and migration, where its overexpression may lead to endothelial damage and promote PANoptosis-related cell death. Additionally, its significant association with M0 macrophages suggests that ITGAM may influence macrophage adhesion and differentiation. This could facilitate the transition of M0 macrophages toward a pro-inflammatory phenotype, further amplifying vascular inflammation and remodeling in PAH; S100A8, a calcium-binding protein, was found in this study to be associated with neutrophils and monocytes. Its involvement in immune cell recruitment and activation suggests a role in amplifying inflammation. Through neutrophils, S100A8 may promote endothelial damage via reactive oxygen species and NETs, while its connection with monocytes highlights its role in driving pro-inflammatory signaling and vascular remodeling (42); CD14, a receptor in monocytes and macrophages, is involved in pathogen recognition and inflammation initiation, where its high expression may exacerbate PAH and atherosclerosis progression, triggering the PANoptosis process through excessive immune activation (43). Additionally, SFRP2, a regulator of the Wnt signaling pathway, is closely related to cardiovascular development and repair (44). Its abnormal expression in PAH and other cardiovascular diseases may influence cell death–related signaling pathways and is potentially associated with PANoptosis-related forms of programmed cell death. These core genes are involved in the regulation of inflammation and immune responses in PAH, suggesting a possible involvement of PANoptosis in the disease's pathogenesis, which warrants further investigation.

Following validation in external datasets, SFRP2 was identified as the most stable hub gene, suggesting its potential role in PAH and fibrosis. Although direct studies on SFRP2 in PAH are limited, its established involvement in the Wnt signaling pathway provides a strong basis for its potential contribution to PAH pathogenesis. The Wnt pathway regulates processes such as vascular remodeling, cell proliferation, and differentiation, and SFRP2 may influence these processes by modulating signaling dynamics within pulmonary vascular cell (45). In fibrotic diseases, including IPF, SFRP2 promotes ECM remodeling by enhancing the activity of MMP-2 and MMP-9, leading to collagen deposition and tissue stiffness. These processes are hallmarks of pulmonary vascular remodeling in PAH. While specific studies on SFRP2 in PAH are lacking, its shared role in fibrosis across tissues suggests a potential contribution to fibrotic changes in pulmonary arterioles (46). Notably, fibrosis in PAH is driven by the activation of PAAFs, which are key mediators of ECM remodeling and vascular stiffening (47). To investigate this, our study focused on PAAFs and revealed, for the first time, that SFRP2 deficiency significantly reduces TGF-β1-induced proliferation and anti-apoptotic activity in PAAFs. These findings highlight the critical role of SFRP2 in fibroblast activation, contributing to ECM deposition and vascular remodeling in PAH. Additionally, SFRP2 interacts with TGF-β signaling to further enhance fibroblast activation and differentiation into myofibroblasts, exacerbating fibrosis and vascular stiffness (48).

SFRP2 has been implicated in a range of vascular diseases, including atherosclerosis, hypertension-induced vascular remodeling, and cardiac fibrosis (49). In hypertension-induced vascular remodeling, SFRP2 expression is associated with increased extracellular matrix deposition and vessel wall thickening, potentially exacerbating vascular stiffness and dysfunction. These findings suggest that SFRP2 may serve as a critical modulator of vascular pathology, further highlighting its relevance in PAH progression. Recent studies suggest that SFRP2 may also be involved in programmed cell death regulation, including pathways related to PANoptosis. While traditionally considered a Wnt pathway modulator, SFRP2 has been linked to apoptotic resistance, inflammation, and immune cell regulation in various disease contexts (50). Notably, PANoptosis, an inflammatory form of cell death integrating pyroptosis, apoptosis, and necroptosis, is known to play a role in chronic inflammatory and fibrotic diseases, including those affecting the lungs and vasculature (51). Emerging evidence indicates that SFRP2 may influence these processes through its effects on immune cell infiltration and inflammatory signaling cascades, particularly via NF-*κ*B and IL-1β, both of which are key regulators of PANoptosis (52). This suggests a potential mechanistic link between SFRP2, PANoptosis, and the progression of PAH. However, the direct role of SFRP2 in PANoptosis within PAH remains to be elucidated and warrants further investigation. In summary, SFRP2 serves as a key regulator of fibroblast-driven fibrosis and vascular dysfunction in PAH through its modulation of Wnt and TGF-β signaling pathways. These findings provide new insights into the role of SFRP2 in PAH pathogenesis and its potential as a therapeutic target.

However, our study has several limitations. Firstly, the sample size is relatively small, and variability across different datasets may introduce potential biases in the results. Therefore, expanding the sample size in future studies is essential to further validate these findings and improve the accuracy of clinical models. Differences in sequencing platforms, patient demographics, and disease subtypes across datasets could lead to batch effects and impact the reproducibility of our findings. Therefore, expanding the sample size in future studies, including multi-center cohort studies, is essential to further validate these findings and improve the accuracy of clinical models. Additionally, the potential impact of sample heterogeneity in human datasets should be acknowledged. PAH is a highly heterogeneous disease influenced by genetic, environmental, and comorbid factors, which may lead to variability in gene expression profiles. Future studies should stratify patients based on relevant clinical parameters, such as PAH etiology, disease severity, and treatment history, to refine biomarker selection and improve model robustness. Secondly, clinical samples were not included in our validation process, which highlights the need for more comprehensive clinical studies to assess the clinical applicability of these findings. SFRP2, as a secreted protein, can be detected in blood samples, making it a promising biomarker for clinical practice. Future research could explore incorporating SFRP2 into a risk score alongside clinical characteristics and other routine biomarkers, such as C-reactive protein, to assess the risk and progression of PAH. Additionally, several confounding factors, including treatment history, disease stage, and comorbidities, such as age, gender, lifestyle, and environmental exposures, may influence SFRP2 expression and its diagnostic potential. These factors should be carefully considered and controlled in future clinical trials. Furthermore, a prospective clinical trial with a larger sample size, comparing a control group with a cohort of already diagnosed PAH patients, will be crucial to confirm the findings of this study. Such a trial will help further validate the diagnostic value of SFRP2 and other biomarkers and provide evidence for their combined use in early screening, risk assessment, and monitoring therapeutic response in PAH. Moreover, while our study primarily explored the role of SFRP2 in PAH-related fibroblast proliferation and apoptosis resistance, further validation is needed to determine whether PANoptosis plays a definitive role in PAH pathogenesis. Although our findings suggest a potential link, additional studies incorporating caspase activation assays, LDH release assays, and inflammasome activation detection are necessary to fully elucidate the involvement of PANoptosis and the regulatory role of SFRP2 in this process. Lastly, future research should also investigate the precise mechanisms by which these characteristic genes impact PAH through PANoptosis regulation. In addition, performing genetic manipulation studies, such as altering SFRP2 expression in animal models, would strengthen the causal relationship between SFRP2 and the pathogenesis of PAH. Despite these limitations, our study offers important insights into PAH diagnosis and treatment strategies, particularly in the context of therapeutic interventions targeting the PANoptosis pathway.

## 5 Conclusion

In conclusion, through a comprehensive analysis of PANoptosis characteristics in PAH, we identify SFRP2 as a robust predictive marker. Knockdown of SFRP2 suppresses the proliferation of pulmonary artery fibroblasts and the progression of apoptosis resistance, suggesting that SFRP2 may serve as a potential therapeutic target for PAH.

## Data Availability

The original contributions presented in the study are included in the article/Supplementary Material, further inquiries can be directed to the corresponding author.
