# Supplementary material for: Identification of a PANoptosis-related gene signature reveals therapeutic potential of SFRP2 in pulmonary arterial hypertension
Source: Front Cardiovasc Med. 2025 Apr 29;12:1521087. doi: 10.3389/fcvm.2025.1521087 (PMC12069314; doi:10.3389/fcvm.2025.1521087)
Supplement: Supplementary file 1 [file Table1.docx]

Supplementary Material

# Supplementary Data

# Supplementary Figures and Tables

**Supplementary Table 1**

| AATF | COA8 | GFRAL | MIR15A | PRKDC | TGFB1 | GSDME | CHMP6 |
| --- | --- | --- | --- | --- | --- | --- | --- |
| ABL1 | COL2A1 | GGCT | MIR16-1 | PRKN | TGFB2 | GZMB | VPS4 |
| ACAA2 | CRADD | GHITM | MIR17 | PRKRA | TGFBR1 | HMGB1 | CHMP1 |
| ACKR3 | CREB3 | GNAI2 | MIR198 | PRODH | THBS1 | IL18 | CHMP5 |
| ACVR1 | CREB3L1 | GNAI3 | MIR21 | PSEN1 | TICAM1 | IL6 | SMPD1 |
| ACVR1B | CRH | GPER1 | MIR210 | PSMD10 | TICAM2 | NOD2 | PYCARD |
| ADORA1 | CRIP1 | GPX1 | MIR221 | PSME3 | TIMM50 | IRF1 | NLRP3 |
| AEN | CSF2 | GRINA | MIR222 | PTEN | TIMP3 | IRF2 | ZBP1 |
| AGT | CSNK2A1 | GSDME | MIR26B | PTGIS | TLR3 | NLRC4 | IL33 |
| AGTR2 | CSNK2A2 | GSK3A | MIR27B | PTH | TLR4 | NLRP1 | FTL |
| AIFM1 | CTH | GSK3B | MIR449A | PTPMT1 | TM2D1 | NLRP2 | SQSTM1 |
| AKT1 | CTNNA1 | GSKIP | MKNK2 | PTPN1 | TMBIM1 | NLRP3 | VDAC2 |
| ANXA6 | CTSC | GSTP1 | MLH1 | PTPN2 | TMBIM6 | NLRP6 | VDAC3 |
| APAF1 | CTTN | GZMB | MLLT11 | PTPRC | TMC8 | NLRP7 | CHMP7 |
| APPL1 | CUL1 | HDAC1 | MMP9 | PTTG1IP | TMEM102 | NOD1 | PGAM5 |
| AR | CUL2 | HERPUD1 | MNT | PYCARD | TMEM109 | PLCG1 | BIRC2 |
| ARHGEF2 | CUL3 | HGF | MOAP1 | QARS1 | TMEM117 | PJVK | BIRC3 |
| ARL6IP5 | CUL4A | HIC1 | MPV17L | RACK1 | TMEM14A | PRKACA | EIF2AK2 |
| ARMC10 | CUL5 | HIF1A | MSH2 | RAF1 | TMEM161A | PYCARD | PLA2G4 |
| ARRB2 | CX3CL1 | HINT1 | MSH6 | RB1 | ZSWIM2 | SCAF11 | DNM1L |
| ASAH2 | CX3CR1 | HIP1 | MSX1 | RB1CC1 | TNFAIP3 | TINAP | SPATA2 |
| ATF3 | CXCL12 | HIP1R | MUC1 | RBCK1 | TNFRSF10A | TIRAP | FAF1 |
| ATF4 | CYLD | HIPK1 | MUL1 | RELA | TNFRSF10B | TP53 | SHARPIN |
| ATM | CYP1B1 | HIPK2 | MYBBP1A | RET | TNFRSF10C | TP63 | NOX2 |
| ATP2A1 | DAB2IP | HMGB2 | NACC2 | RFFL | TNFRSF12A | AIM2 | USP21 |
| ATP2A3 | DAP | HMOX1 | NANOS3 | RHOT1 | TNFRSF1A | GSDMA | PARP1 |
| ATP5IF1 | DAP3 | HNRNPK | NBN | RHOT2 | TNFRSF1B | GLUD1 |  |
| AVP | DAPK1 | HRAS | NCK1 | RIPK1 | TNFRSF25 | GLUD2 |  |
| BAD | DAPK2 | HRK | NCK2 | RIPK3 | TNFSF10 | ALOX15 |  |
| BAG3 | DAPK3 | HSPA1A | NDUFA13 | RNF183 | TNFSF12 | FTH1 |  |
| BAG5 | DAPL1 | HSPA1B | NDUFS3 | RNF186 | TOPORS | PYG |  |
| BAG6 | DAXX | HSPB1 | NFATC4 | RNF34 | TP53 | CAPN1 |  |
| BAK1 | DBH | HTRA2 | NFE2L2 | RNF41 | TP53BP2 | CASP1 |  |
| BAX | DCC | HTT | NGF | RPL11 | TP63 | GLNA |  |
| BBC3 | DDIAS | HYAL2 | NGFR | RPL26 | TP73 | BAX |  |
| BCAP31 | DDIT3 | HYOU1 | NKX3-1 | RPS27L | TPD52L1 | BCL2 |  |
| BCL10 | DDIT4 | ICAM1 | NLE1 | RPS3 | TPT1 | FADD |  |
| BCL2 | DDX3X | IFI16 | NME5 | RPS6KB1 | TRADD | RIPK1 |  |
| BCL2A1 | DDX47 | IFI27 | NMT1 | RPS7 | TRAF1 | CHMP4C |  |
| BCL2L1 | DDX5 | IFI27L1 | NOC2L | RRP8 | TRAF2 | TNFRSF1A |  |
| BCL2L10 | DEDD | IFI27L2 | NOG | RTKN2 | TRAF7 | TRADD |  |
| BCL2L11 | DEDD2 | IFI6 | NOL3 | RTL10 | TRAP1 | TRAF2 |  |
| BCL2L12 | DELE1 | IFNB1 | NONO | S100A8 | TRIAP1 | PPIA |  |
| BCL2L14 | DEPTOR | IFNG | NOS3 | S100A9 | TRIB3 | CAPN2 |  |
| BCL2L2 | DIABLO | IGF1 | NOX1 | SCG2 | TRIM32 | HSP90A |  |
| BCL3 | DIDO1 | IKBKE | NR4A2 | SCN2A | TRIM39 | FAS |  |
| BCLAF1 | DNAJA1 | IL12A | NUPR1 | SCRT2 | TXNDC12 | TNFSF6 |  |
| BDKRB2 | DNAJC10 | IL19 | OPA1 | SELENOK | TYROBP | TNFRSF6 |  |
| BDNF | DNM1L | ZNF385B | P2RX4 | SELENOS | UACA | CASP8 |  |
| BECN1 | DPF2 | ZNF622 | P2RX7 | SENP1 | UBB | JNK |  |
| BID | DYRK2 | IL2 | P4HB | SEPTIN4 | UBE2K | JAK2 |  |
| BIK | E2F1 | IL20RA | PAK2 | SERINC3 | UBE4B | CAMK2 |  |
| BIRC6 | E2F2 | IL33 | PAK5 | SERPINE1 | UBQLN1 | FASLG |  |
| BLOC1S2 | EDA2R | IL4 | PARK7 | SFN | UMOD | IFNG |  |
| BMF | EIF2AK3 | IL6R | PARP1 | SFPQ | UNC5B | STAT3 |  |
| BMP4 | ELL3 | IL7 | PARP2 | SFRP1 | URI1 | IRF9 |  |
| BMP5 | ENO1 | INCA1 | PAWR | SFRP2 | USP28 | TNFSF10 |  |
| BMPR1B | EP300 | ING2 | PCGF2 | SGMS1 | USP47 | TNFRSF10A | |
| BNIP3 | EPHA2 | ING5 | PDCD10 | SGPL1 | VDAC2 | TNFRSF10B | |
| BNIP3L | EPO | INHBA | PDCD5 | SGPP1 | VNN1 | CFLAR |  |
| BOK | ERBB3 | INHBB | PDCD6 | SH3RF1 | WDR35 | XIAP |  |
| BRCA1 | ERCC6 | INS | PDIA3 | SHH | WNT4 | BID |  |
| BRCA2 | ERN1 | ITGA6 | PDK1 | SHISA5 | WWOX | AIFM1 |  |
| BRSK2 | ERN2 | ITGAM | PDK2 | SIAH1 | XBP1 | TRPM7 |  |
| BTK | ERO1A | ITGAV | PDPK1 | SIAH2 | YAP1 | IFNAR1 |  |
| CAAP1 | ERP29 | ITM2C | PDX1 | SIRT1 | YBX3 | IFNAR2 |  |
| CASP1 | EYA1 | ITPR1 | PEA15 | SIVA1 | YWHAB | IFNGR1 |  |
| CASP10 | EYA2 | ITPRIP | PELI3 | SKIL | YWHAE | IFNGR2 |  |
| CASP12 | EYA3 | IVNS1ABP | PERP | SLC25A5 | YWHAG | TLR3 |  |
| CASP2 | EYA4 | JAK2 | PF4 | SLC35F6 | YWHAH | TIRP |  |
| CASP3 | FADD | JMY | PHIP | SLC9A3R1 | YWHAQ | IFNA |  |
| CASP4 | FAF1 | JUN | PHLDA3 | SMAD3 | YWHAZ | IFNB |  |
| CASP5 | FAIM | KDM1A | PIAS4 | SNAI1 | ZC3HC1 | TRIF |  |
| CASP8 | FAIM2 | KITLG | PIDD1 | SNAI2 | ZDHHC3 | VDAC1 |  |
| CASP8AP2 | FAM162A | KRT18 | PIH1D1 | SNW1 | ZMYND11 | SLC25A4S |  |
| CASP9 | FAS | KRT8 | PIK3R1 | SOD1 | ZNF205 | PPID |  |
| CAV1 | FASLG | LCK | PINK1 | SOD2 | ZNF385A | CYLD |  |
| CCAR2 | FASTK | LGALS12 | PLAGL2 | SORT1 | BAK1 | RIPK3 |  |
| CCK | FBH1 | LGALS3 | PLAUR | SP100 | BAX | MLKL |  |
| CD14 | FBXW7 | LRRK2 | PLEKHF1 | SRC | CASP1 | TRAF5 |  |
| CD24 | FEM1B | LTBR | PLSCR3 | SRPX | CASP3 | TLR4 |  |
| CD27 | FGA | LY96 | PMAIP1 | SST | CASP4 | RBCK1 |  |
| CD28 | FGB | MADD | PML | SSTR3 | CASP5 | HMGB1 |  |
| CD38 | FGF10 | MAEL | POLB | ST20 | CASP6 | JAK1 |  |
| CD3E | FGFR1 | MAGEA3 | POU4F1 | STK11 | CASP8 | JAK3 |  |
| CD44 | FGFR3 | MAP2K5 | POU4F2 | STK24 | CASP9 | TYK2 |  |
| CD5 | FGG | MAP3K5 | PPARD | STK25 | CHMP2A | STAT1 |  |
| CD70 | FHIT | MAPK7 | PPIA | STK3 | CHMP2B | STAT2 |  |
| CD74 | FIGNL1 | MAPK8 | PPIF | STK4 | CHMP3 | STAT4 |  |
| CDIP1 | FIS1 | MAPK8IP1 | PPM1F | STRADB | CHMP4A | STAT5A |  |
| CDKN1A | FNIP2 | MAPK8IP2 | PPP1CA | STX4 | CHMP4B | STAT5B |  |
| CDKN2D | FXN | MAPK9 | PPP1R13B | STYXL1 | CHMP4C | STAT6 |  |
| CEBPB | FYN | MARCHF7 | PPP1R15A | SYVN1 | CHMP6 | H2A |  |
| CFLAR | FZD9 | MAZ | PPP2R1B | TAF9 | CHMP7 | TNFAIP3 |  |
| CHAC1 | G0S2 | MCL1 | PPP3CC | TAF9B | CYCS | RNF31 |  |
| CHCHD10 | GABARAP | MDM2 | PPP3R1 | TCF7L2 | ELANE | CHMP2A |  |
| CHEK2 | GATA1 | MELK | PRDX2 | TERT | GPX4 | CHMP2B |  |
| CIB1 | GATA4 | MFF | PRELID1 | TFDP1 | GSDMB | VPS24 |  |
| CIDEB | GCLM | MIF | PRKCA | TFDP2 | GSDMC | CHMP4A |  |
| CLU | GDNF | MIR132 | PRKCD | TFPT | GSDMD | CHMP4B |  |

**Supplementary Table 2**

| **Gene** | **Sequence (5'-3')** |
| --- | --- |
| ITGAM F | AGCAGGGATCATTCGCTACG |
| ITGAM R | GGTACTTCCTGTCTGGGTGC |
| S100A8 F | TGGCAACTGAACTGGAGAAGG |
| S100A8 R | CCCTGTAGAGGGCATGGTGA |
| CD14 F | GTCCAGCGCTTTTCTATGCG |
| CD14 R | GTTATACGCCTCCGACTGGG |
| SFRP2 F | CAGAGGAAGCTCCCAAGGTG |
| SFRP2 R | GACACCCCGTTCAGCTTGTA |

## Supplementary Figures

**
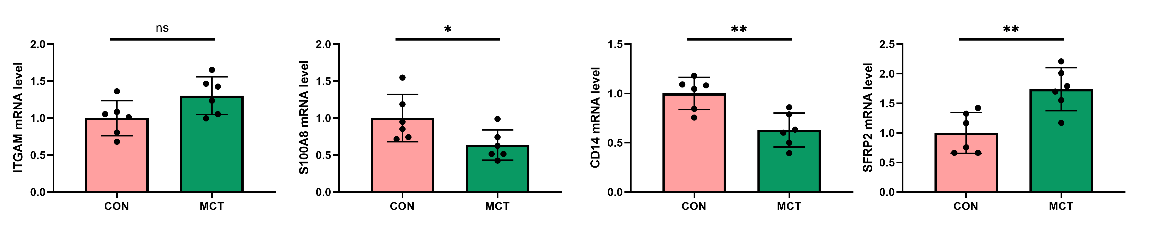
**

**Supplementary Figure 1.** The PAH rat model was constructed, and the expression of the four characteristic genes was experimentally detected by qRT-PCR. PAH, pulmonary ateria hypertension; Data are presented as mean ± SEM. **p* < 0.05,***p* < 0.01


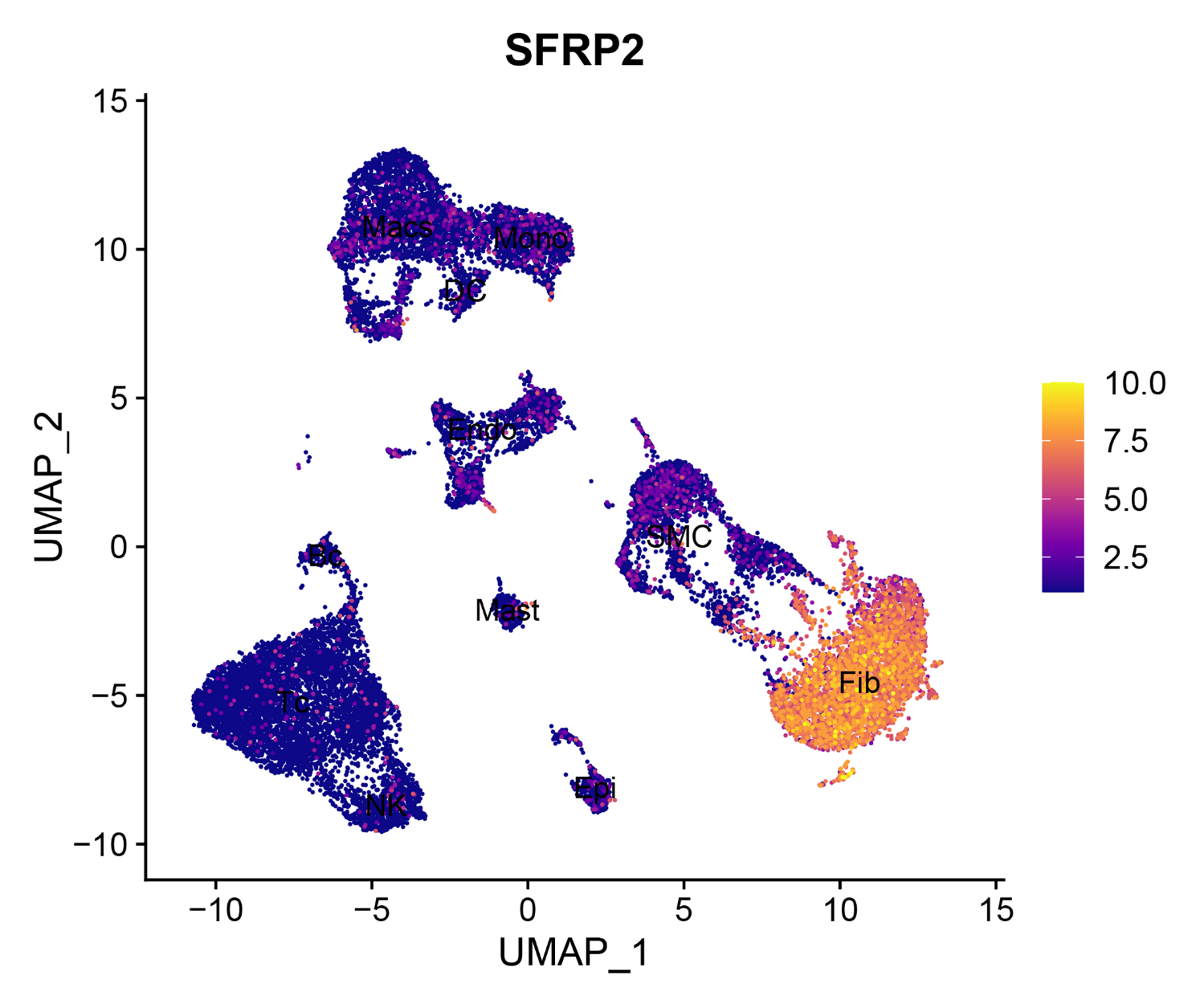


**Supplementary Figure 2.** SFRP2 is expressed in fibroblasts of the pulmonary artery.
